# Supplementary material for: Climate-driven tradeoffs between landscape connectivity and the maintenance of the coastal carbon sink
Source: Nat Commun. 2023 Mar 13;14:1137. doi: 10.1038/s41467-023-36803-7 (PMC10011419; doi:10.1038/s41467-023-36803-7)
Supplement: Supplementary file 1 — Supplementary Information [file 41467_2023_36803_MOESM1_ESM.pdf]

Climate-driven tradeoffs between landscape connectivity and the maintenance of the coastal carbon sink

SUPPLEMENTARY INFORATION

Kendall Valentine*[1,2], Ellen R. Herbert[1,3], David C. Walters[1,4], Yaping Chen[1], Alexander J. Smith[1], and Matthew L. Kirwan[1]

[1]Virginia Institute of Marine Science, College of William and Mary, Gloucester Point, VA, USA

[2]School of Oceanography, University of Washington, Seattle, WA, USA

[3]Ducks Unlimited, Memphis, TN, USA

[4] U.S. Geological Survey Eastern Ecological Science Center, Laurel, MD, USA

*corresponding author, kvalent@uw.edu

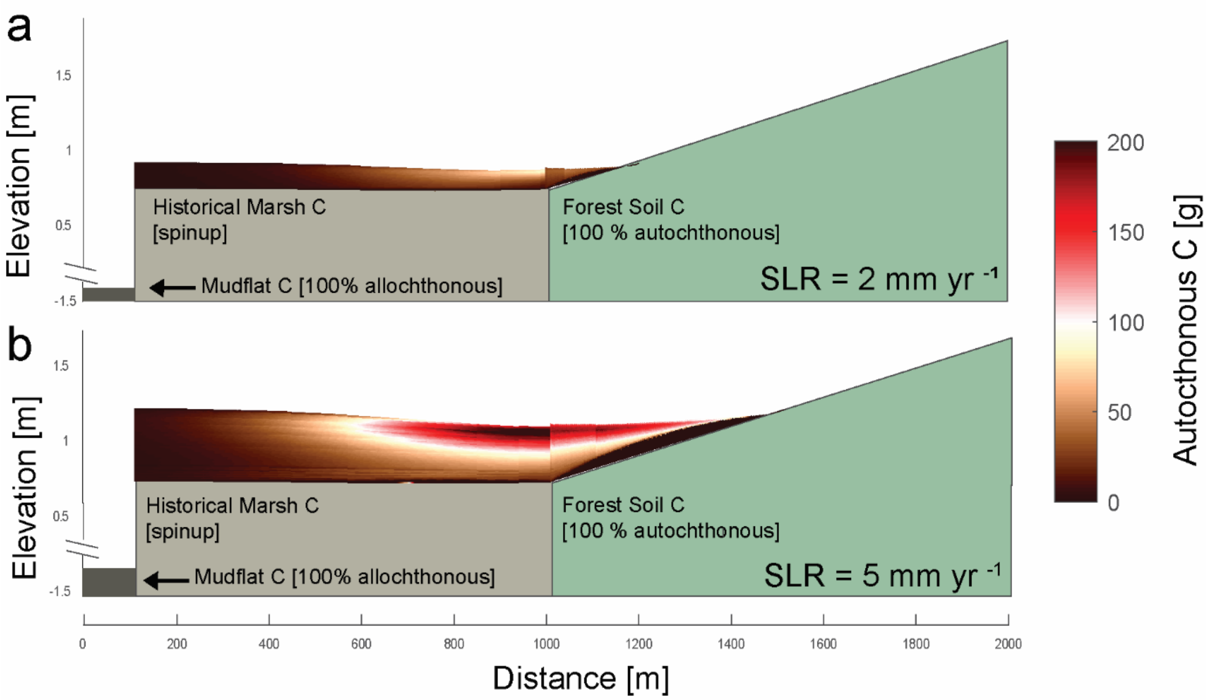

Supplementary Figure 1. Autochthonous carbon after the coastal transect was subjected to low (a) and moderate (b) rates of sea level rise (SLR). Model experiments were conducted under a 50 mg L^{-1} sediment supply and a 1.4 m tidal range. Color shadings along scale on right indicate the amount of autochthonous carbon [g]. Underlying stratigraphy was generated during the model spinup. x-axis distance is relative to initial shoreline position and y-axis is relative to initial sea level.

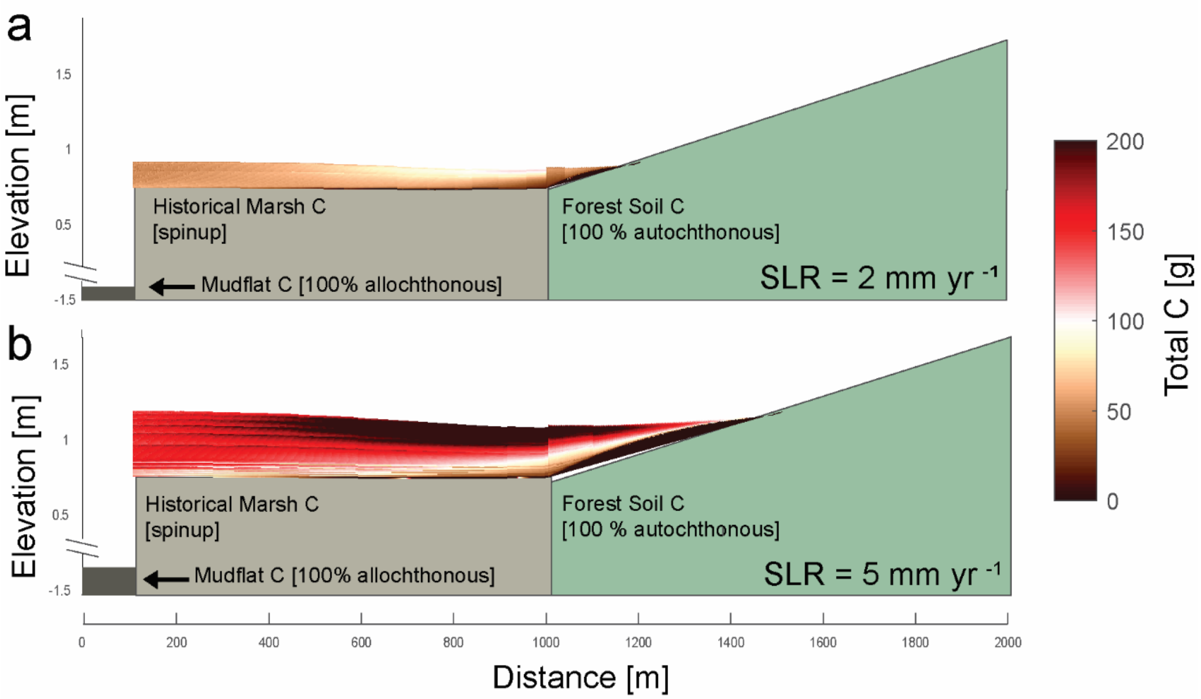

Supplementary Figure 2. Total Carbon after the coastal transect was subjected to low (a) and moderate (b) rates of sea level rise (SLR). Model experiments were conducted under a 50 mg L^{-1} sediment supply and a 1.4 m tidal range. Color shadings along scale on right indicate the total amount of carbon [g]. Underlying stratigraphy was generated during the model spinup. x-axis distance is relative to initial shoreline position and y-axis is relative to initial sea level.

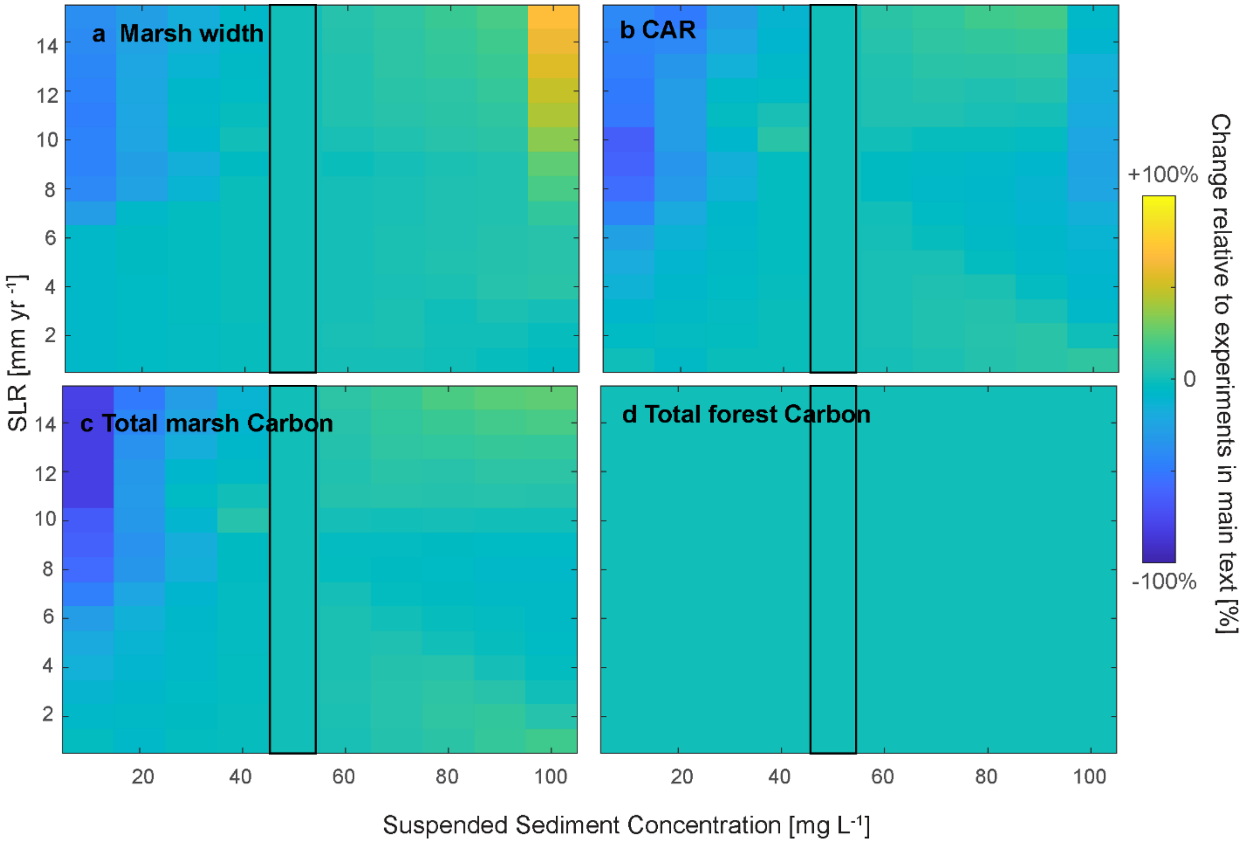

Supplementary Figure 3. Relative change in marsh width (a), carbon accumulation rate (b), total marsh carbon (c), and total forest carbon (d) for a range of suspended sediment concentrations and sea level rise (SLR) rates. Relative change was calculated as the difference between model runs presented in the main text (Figs. 3a-4, SSC=50 mg L^{-1}, SLR = 1-15 mm yr^{-1}) and model runs presented here (SSC=10-100 mg L^{-1}, SLR = 1-15 mm yr^{-1}). The vertical box in each figure indicates the model runs in the main text.

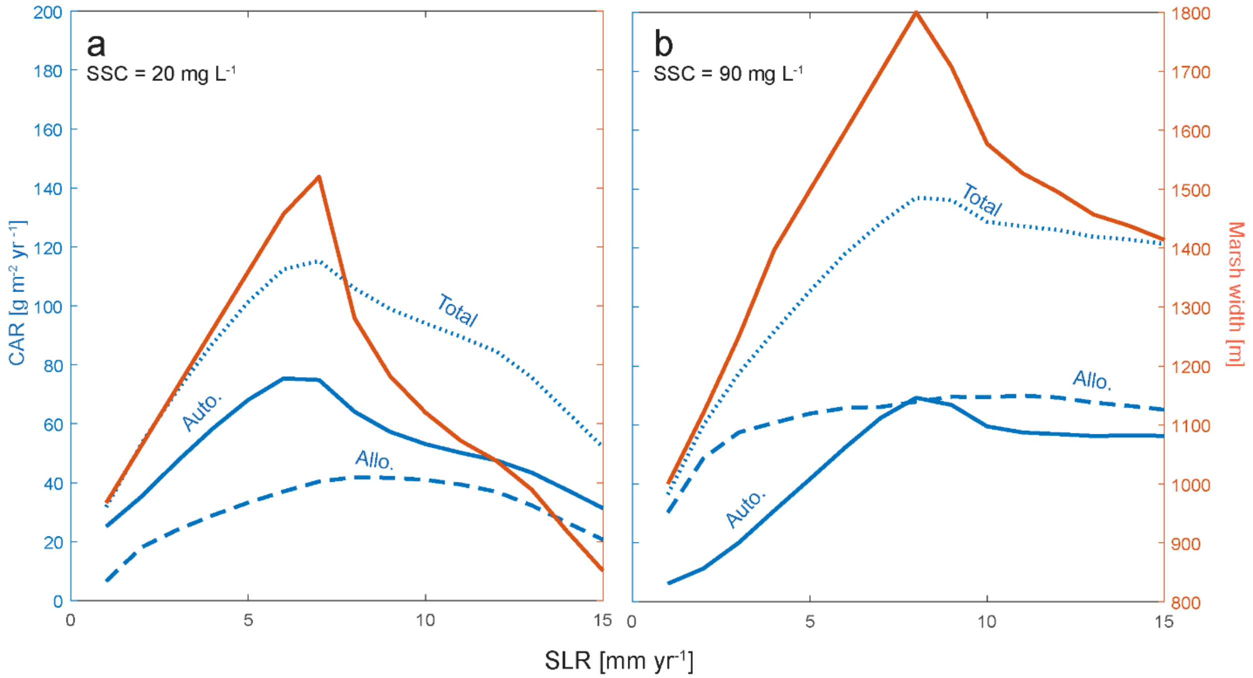

Supplementary Figure 4. Carbon accumulation rate (CAR) and marsh width for suspended sediment concentration (SSC) =20 mg L^{-1} (a) and SSC=90 mg L^{-1} (b). While the values of CAR and marsh width change depending on the suspended sediment concentration, the general patterns observed remain consistent, with a peak in all metrics (except allochthonous C) at intermediate rates of sea level rise (SLR).

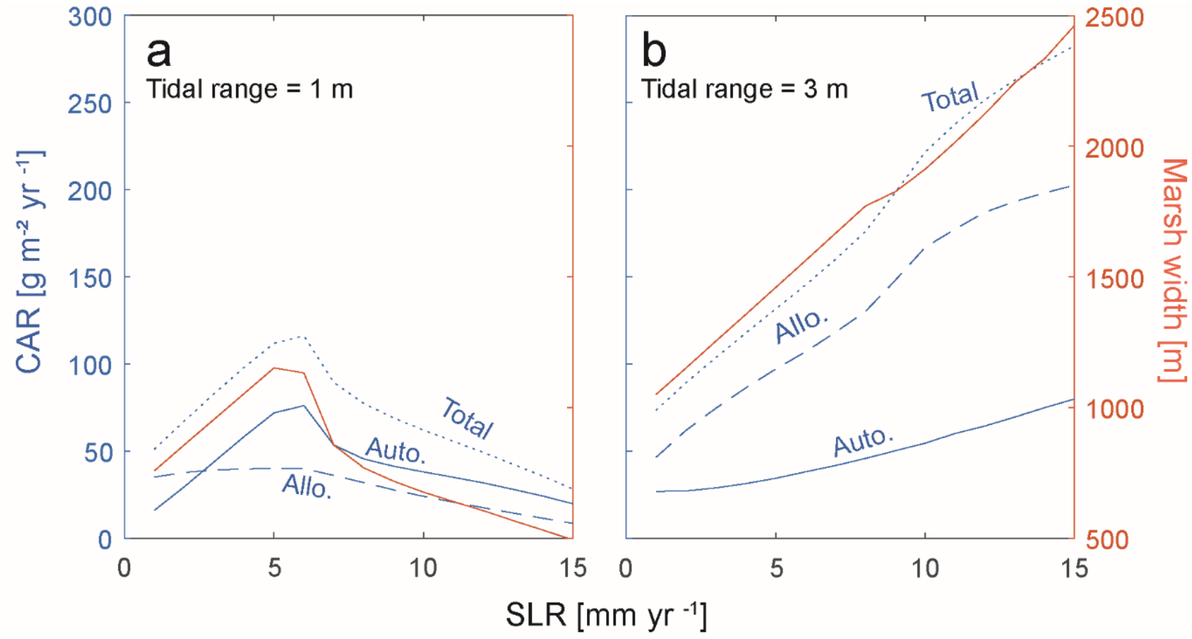

Supplementary Figure 5. Carbon accumulation rate (CAR) and marsh width for tidal range = 1 m (a) and tidal range = 3 m (b). With a small tidal range, CAR and marsh width peak synchronously at an intermediate rate of sea level rise (SLR). At a higher tidal range, all metrics continue to increase with SLR because the marsh platform started at a higher elevation and drowning did not occur.

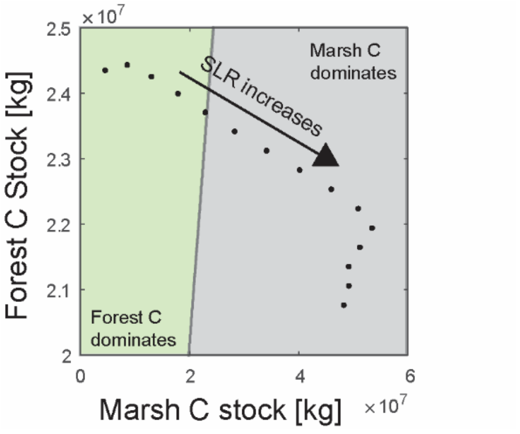

Supplementary Figure 6. Balance between forest carbon (C) and marsh C in the coastal landscape. As sea level rise (SLR) rates increase, the landscape carbon stock transitions from dominantly forest biomass to dominantly marsh soil carbon. Each dot represents one model run with incremental increases in SLR rate.

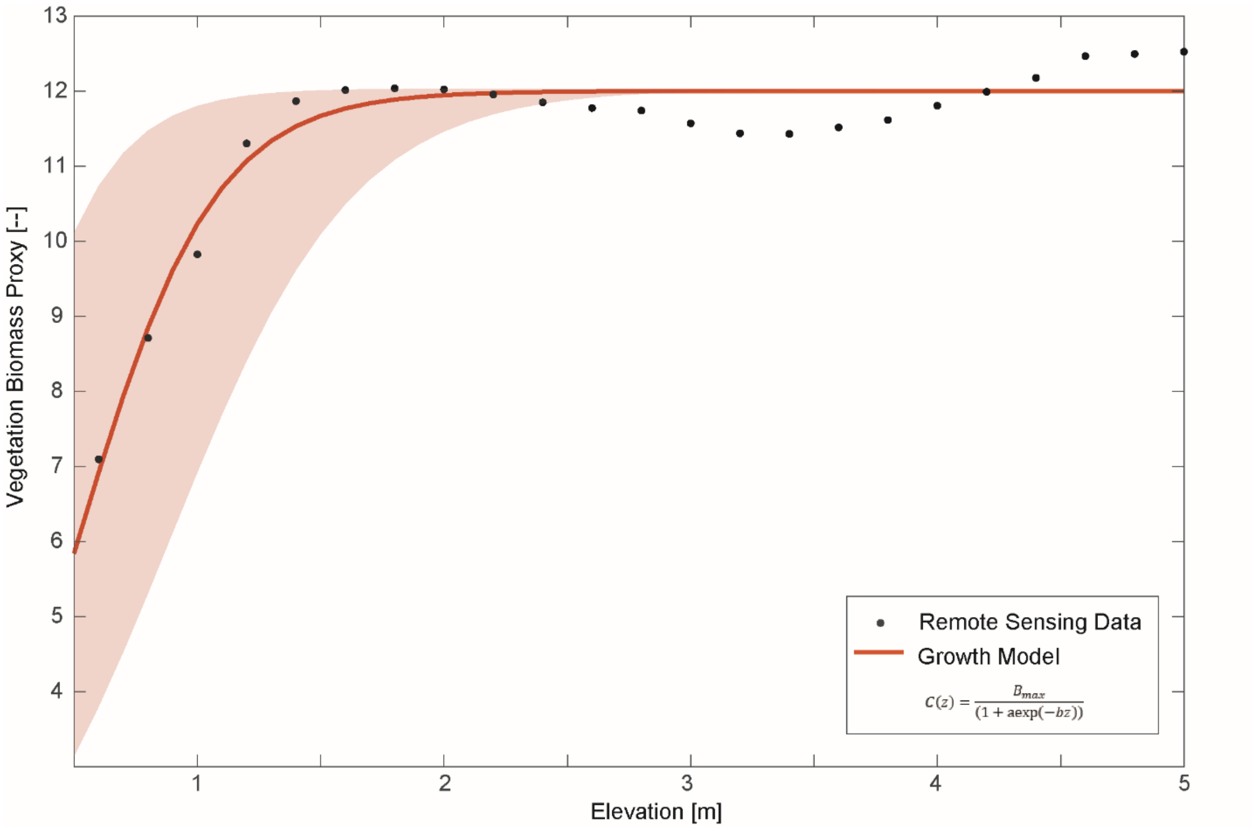

Supplementary Figure 7. Vegetation biomass (aboveground) changes as a function of elevation across the coastal landscape. The datapoints represent average biomass by elevation. The vegetation biomass is converted from remotely-sensed NDVI according to the biomass-NDVI function[1] generated in the Chesapeake Bay region using Landsat images (2016-2020). This data was fit to the form of a logistic model (red line) (95% CI in red envelope, $R^2=0.93$).

Supplementary Table 1: Model parameters. The data sources for all parameters developed for this model are listed in the column named as Source.

Symbol	Parameter	Value	Units	Source
k_e	Lateral erodibility coefficient	0.16	$m\ s^{-1}\ (W\ m^{-1})^{-1}$	Mariotti and Carr 2014[2]
γ	Water specific weight	9800	$N\ m^{-3}$	Mariotti and Carr 2014[2]
w_{sm}	Settling velocity – marsh	0.05	$mm\ s^{-1}$	Mudd et al. 2009[3]
w_{sf}	Settling velocity – bay	0.5	$mm\ s^{-1}$	Mariotti and Carr 2014[2]
P	Tidal period	12.5	hours	Semidiurnal tide
B_{max}	Peak marsh biomass	2500	$g\ m^{-2}$	Morris et al. 2002[4]
k_a	Lateral progradation coefficient	2	dimensionless	Mariotti and Carr 2014[2]
Λ	Sediment decay coefficient	-0.0031	m^{-1}	Kirwan et al. 2016[5]
τ_{cr}	Critical shear stress for erosion	0.1	Pa	Mariotti and Carr 2014[2]
Wind speed	Wind speed	6	$m\ s^{-1}$	Kirwan et al. 2016[5], Mariotti and Carr 2014[2]
Tidal amplitude	Tidal amplitude	0.7	m	Mesotidal
ρ_s	Mineral bulk density	2000	$kg\ m^{-3}$	Morris et al. 2016[6]
ρ_o	Organic matter bulk density	85	$kg\ m^{-3}$	Morris et al. 2016[6]
λ	Bay bottom erodibility coefficient	0.001	dimensionless	Mariotti and Carr 2014[2]
d_{min}	Minimum depth for marsh plant growth	0	m	Kirwan et al. 2016[5]
d_{max}	Maximum depth for marsh plant growth	0.5204	m	McKee and Patrick 1988[7]
m_u	Depth below which decomposition goes to zero	0.4	m	Rietl et al. 2021[8]
m_k	Coefficient of decomposition in the marsh	0.1	dimensionless	Rietl et al. 2021[8]
$B_{max,forest}$	Forest biomass maximum	5000	$g\ m^{-2}$	Empirical, Fig. S1 (Chen and Kirwan, 2022) [1]

$C_{0,agb}$	Tree biomass value at marsh-forest boundary	1000	g m^{-2}	Empirical, Fig. S1 (Chen and Kirwan, 2022) [1]
b	Tree growth rate	2	m^{-1}	Empirical, Fig. S1 (Chen and Kirwan, 2022) [1]
$C_{0,soil}$	Background carbon accumulation in forest soils	0.0001	g m^{-2}	Empirical, Smith and Kirwan 2022 [1]
C_{wet}	Carbon layer from wetted soils	5	g m^{-2}	Empirical, Smith and Kirwan 2022 [1]
b_{soil}	Decay constant	2	m^{-1}	Empirical, Smith and Kirwan 2022[1]
m	Upland slope	0.001	dimensionless	Hussein 2009[9]

Supplementary References

1. Chen, Y. & Kirwan, M. L. Climate-driven decoupling of wetland and upland biomass trends on the mid-Atlantic coast. *Nat. Geosci.* **15**, 913–918 (2022).
2. Mariotti, G. & Carr, J. Dual role of salt marsh retreat: Long-term loss and short-term resilience. *Water Resour. Res.* **50**, 2963–2974 (2014).
3. Mudd, S. M., Howell, S. M. & Morris, J. T. Impact of dynamic feedbacks between sedimentation, sea-level rise, and biomass production on near-surface marsh stratigraphy and carbon accumulation. *Estuar. Coast. Shelf Sci.* **82**, 377–389 (2009).
4. Morris, J. T., Sundareshwar, P. V., Nietch, C. T., Kjerfve, B. & Cahoon, D. R. Responses of Coastal Wetlands to Rising Sea Level. *Ecology* **83**, 2869–2877 (2002).
5. Kirwan, M. L., Walters, D. C., Reay, W. G. & Carr, J. A. Sea level driven marsh expansion in a coupled model of marsh erosion and migration. *Geophys. Res. Lett.* **43**, 4366–4373 (2016).
6. Morris, J. T. *et al.* Contributions of organic and inorganic matter to sediment volume and accretion in tidal wetlands at steady state. *Earths Future* **4**, 110–121 (2016).
7. McKee, K. L. & Patrick, W. H. The relationship of smooth cordgrass (Spartina alterniflora) to tidal datums: A review. *Estuaries* **11**, 143–151 (1988).

8. Rietl, A. J., Megonigal, J. P., Herbert, E. R. & Kirwan, M. L. Vegetation Type and Decomposition Priming Mediate Brackish Marsh Carbon Accumulation Under Interacting Facets of Global Change. *Geophys. Res. Lett.* **48**, e2020GL092051 (2021).
9. Hussein, A. H. Modeling of Sea-Level Rise and Deforestation in Submerging Coastal Ultisols of Chesapeake Bay. *Soil Sci. Soc. Am. J.* **73**, 185–196 (2009).
